# Novel Mutation in GALT Gene in Galactosemia Patient with Group B Streptococcus Meningitis and Acute Liver Failure ^†^

**DOI:** 10.3390/medicina55040091

**Published:** 2019-04-04

**Authors:** Alina Grama, Ligia Blaga, Alina Nicolescu, Călin Deleanu, Mariela Militaru, Simona Sorana Căinap, Irina Pop, Georgia Tita, Claudia Sîrbe, Otilia Fufezan, Mihaela Adela Vințan, Romana Vulturar, Tudor Lucian Pop

**Affiliations:** 1 Second Pediatric Clinic, Department of Mother and Child, University of Medicine and Pharmacy “Iuliu Hațieganu”, 400012 Cluj-Napoca, Romania; gramaalina16@yahoo.com (A.G.); cainap.simona@gmail.com (S.S.C.); irina26pop@gmail.com (I.P.); tartamus.georgia@gmail.com (G.T.); claudia.sirbe@yahoo.com (C.S.); 2 Discipline of Neonatology, Department of Mother and Child, University of Medicine and Pharmacy “Iuliu Hațieganu”, 400012 Cluj-Napoca, Romania; blagaligia@yahoo.com; 3 NMR Laboratory, “Petru Poni” Institute of Macromolecular Chemistry, Romanian Academy of Sciences, 700487 Iaşi, Romania; alina@icmpp.ro (A.N.); calin.deleanu@yahoo.com (C.D.); 4 “Costin D. Neniţescu” Institute of Organic Chemistry, Romanian Academy of Sciences, 060023 Bucharest, Romania; 5 Medical Genetics, Department of Molecular Sciences, University of Medicine and Pharmacy “Iuliu Hațieganu”, 400012 Cluj-Napoca, Romania; dr.mariela.militaru@gmail.com; 6 Genetic Center Cluj-Napoca, 400363 Cluj-Napoca, Romania; 7 Radiology Department, Children’s Emergency Clinical Hospital, 400378 Cluj-Napoca, Romania; otilia.fufezan@gmail.com; 8 Pediatric Neurology Clinic, Children’s Emergency Clinical Hospital, Department of Neurosciences, University of Medicine and Pharmacy “Iuliu Hațieganu”, 400012 Cluj-Napoca, Romania; adelavintan@yahoo.com; 9 Department of Molecular Sciences, University of Medicine and Pharmacy “Iuliu Hațieganu”, 400012 Cluj-Napoca, Romania; 10 Cognitive Neuroscience Laboratory, Department of Psychology, Babeş-Bolyai University, 400084 Cluj-Napoca, Romania; 11 Center of Expertise for Pediatric Liver Rare Disorders, Children’s Emergency Clinical Hospital, 400177 Cluj-Napoca, Romania; 12 Imogen Medical Institute, 400012 Cluj-Napoca, Romania

**Keywords:** galactosemia, group B streptococcus meningitis, acute liver failure, nuclear magnetic resonance (NMR) spectroscopy, GALT mutations

## Abstract

Classic galactosemia is an autosomal recessive disorder caused by the deficiency of the enzyme galactose-1-phosphate uridyltransferase (GALT) involved in galactose metabolism. Bacterial infections are a known cause of early morbidity and mortality in children with classic galactosemia. The most common agent is *Escherichia coli*, but in rare situations, other bacteria are incriminated. We report a case of a three-week-old female patient with galactosemia, who presented with *Group B Streptococcus* (GBS) meningitis/sepsis. She received treatment with antibiotics, supportive therapy, and erythrocyte transfusion, but after a short period of improvement, she presented acute liver failure with suspicion of an inborn error of metabolism. Rapid nuclear magnetic resonance (NMR) spectroscopy from urine showed highly elevated values of galactose and galactitol. Under intensive treatment for acute liver failure and with a lactose-free diet, her clinical features and laboratory parameters improved considerably. Genetic testing confirmed compound heterozygous status for GALT mutations: c.563 A>G [p.Q188R] and c. 910 C>T, the last mutation being a novel mutation in GALT gene. In countries without an extensive newborn screening program, a high index of suspicion is necessary for early diagnosis and treatment of galactosemia.

## 1. Introduction

Galactose-related disorders are autosomal recessive disorders caused by the deficiency of one of the enzymes involved in galactose metabolism. There are three types of enzymatic deficiencies: galactose-1-phosphate uridyltransferase (GALT) deficiency (classical galactosemia), galactokinase (GALK) deficiency and galactose-6-phosphate epimerase (GALE) deficiency. The diagnosis of galactosemia should be considered in countries where newborn screening does not include galactosemia, in all newborns or infants with any of the following features: failure to thrive, jaundice, hepatomegaly, splenomegaly, poor feeding, vomiting, lethargy, hypoglycemia, convulsions, full fontanelle, cataract, excessive bruising, bleeding diathesis, and renal tubular acidosis [1].

This disorder should be urgently diagnosed because early exclusion of any source of lactose and galactose in the children’s diet produces a rapid clinical improvement of liver damage, jaundice resolves within days, cataracts may clear, kidney functions return to normal, and liver cirrhosis may be prevented [1,2].

## 2. Case Presentation

We report a case of a three-week-old female with classical galactosemia who presented with *Group B Streptococcus* (GBS) meningitis and acute liver failure (ALF) [3]. She was born at 39 weeks of gestation, by cesarean section for maternal indication (uterine scar). She presented premature rupture of membranes for 30 h. The pregnancy was normal, and it was periodically monitored at the local hospital. In the third trimester of pregnancy, the mother had two episodes of vulvovaginitis treated with local antibiotics. There was no consanguinity of the parents and her family has no history of inherited disease.

At birth, the baby’s weight was 3700 g, height was 50 cm and Apgar score was 10. She presented intense jaundice on the second day of life for which she received several phototherapy sessions. She started breastfeeding on her third day of life. Six days after birth, the mother and child were discharged from the regional hospital. A few days later, she became lethargic, with intense jaundice and signs of dehydration. She was initially admitted to the Neonatology Department, presenting jaundice, hepatosplenomegaly, anemia, thrombocytopenia, and high level of bilirubin levels (total bilirubin 27.84 mg/dL, conjugated bilirubin 8.68 mg/dL). Her acute-phase reactants had increased, and blood culture and culture from the cerebrospinal fluid (CSF) were positive for GBS. Cerebral magnetic resonance imaging (MRI) described specific meningitis lesions and cerebral edema. She received antibiotic treatment (ampicillin associated with gentamycin, then meropenem associated with vancomycin), fluconazole intravenous, albumin intravenous infusion, and erythrocyte transfusion (due to severe anemia). Due to the severe evolution with aggravating liver disease (INR 1.6, not corrected with vitamin K), after a few days, she was suspected of an inborn error of metabolism. Urine was collected for rapid urinary nuclear magnetic resonance (NMR) spectroscopy, which was performed with our adapted protocol previously described [4,5] for several metabolic studies using a Bruker Avance III 400 MHz spectrometer, equipped with gradients on the z-axis. The one-step-blood-ammonia-measurement (using the micro diffusion method with reflection registration at λ 635 nm) identified an increased value of 177 µg/dL (normal values for ammonia using this method are less than 54 µg/dL). According to the literature, these moderate increased values were considered just secondary modifications of hepatic dysfunctions, and after a few hours, the results of the urinary NMR spectroscopy showed highly elevated concentrations of galactose (79,839 mmol/mol creat.) and galactitol (41,734 mmol/mol creat.). The patient was transferred to our pediatric hospital (2nd Pediatric Clinic, Cluj-Napoca, Romania) with signs of encephalopathy (second degree coma), jaundice, hepatosplenomegaly (liver at 4 cm, spleen at 3 cm below costal margin), ascites, petechiae and bleeding at the sites of venous puncture. The initial laboratory parameters in our unit revealed increased transaminases (AST 128 U/L, ALT 57 U/L), high bilirubin levels (total bilirubin 20.19 mg/dL, and increased conjugated bilirubin 15.65 mg/dL), hypoalbuminemia (2.6 g/dL), high ferritin level (2,156 ng/mL) and prolonged prothrombin time (23 s) with INR 2.8. She also had moderate hemolytic anemia (hemoglobin 9.2 g/dL) with negative Combs test, leukocytosis (23,500 /mm³ with neutrophilia 89%) and thrombocytopenia (56,000/mm³). Unfortunately, erythrocyte transfusion was given before the suspicion of galactosemia and the measurement of GALT enzyme activity in erythrocytes was not performed. The ophthalmologic examination revealed “oil drop” cataract, which is common in classic galactosemia [6]. Genetic testing for GALT gene confirmed the presence of two mutations as compound heterozygous status: one at exon 6 of the GALT gene, c.563 A>G [p. Q188R] and another one on exon 10 (not yet described in galactosemia), c. 910 C>T. GALT gene was analyzed by PCR and bidirectional sequencing of the whole coding region and intron-exon splicing junctions. MLPA was used for detection of the deletions and duplications of one or more exons. The obtained sequences were compared with the sequence of reference ENST00000378842. The genetic test of the parents was not performed due to their refusal.

Based on the clinical presentation and laboratory parameters, the final diagnosis made was classic galactosemia with GBS meningitis and ALF. She was treated with high doses of antibiotics (meropenem associated with vancomycin), intravenous immunoglobulins (IVIG), albumin intravenous infusion, furosemide, and spironolactone. As she had presented with cerebral edema, she received mannitol, dexamethasone and furosemide. Her diet was immediately changed from breastfeeding to exclusive parenteral nutrition with glucose/arginine infusion (in the first days in our clinic), and then enteral nutrition with soy milk.

Two weeks later, her clinical features and laboratory parameters improved considerably. The level of galactose and galactitol excretion in urine decreased. Recent follow up, at two years of age, showed normal physical and neurological development, normal laboratory parameters, and the absence of cataract (on ophthalmologic slit-lamp examination).

## 3. Discussion

For infants, milk is the main dietary source of galactose. It contains lactose, a disaccharide that is hydrolyzed in the small bowel in glucose and galactose. The galactose is absorbed in the intestinal mucosa and is metabolized to glucose and stored as glycogen in the liver and skeletal muscles, and in very small quantities in kidneys, and other tissues or cells. Initially galactose is metabolized in galactoso-1-phosphate (Gal-1-P) in the presence of galactokinase (GALK). In the second step, Gal-1-P is metabolized in glucoso-1-phosphate under the action of UDP-glucose-galactoso-1-phosphatidyl transferase (GALT). Then, glucoso-1-phosphat, in the presence of UDP-galactoso-4-epimerase (GALE) is metabolized in UDP-glucose, which plays an important role in glycogenesis [1,2]. Depending on the enzyme that is deficient (GALK, GALT or GALE), there are three types of diseases [2].

Classic galactosemia (type 1) represents the most severe form of the disease and is caused by the deficiency of galactose-1-phosphate uridyltransferase (GALT). The incidence is 1 case per 20,000–50,000 newborns and is the most common form in the European population. The absence of GALT or reducing its activity causes the accumulation of galactose, Gal-1-P, galactitol or galactonate in the liver, brain, kidney or other organs [2,7].

Numerous mutations of GALT gene have been identified [8]. The most frequent mutation is Q188R, which determines the absence of enzymatic activity and implicitly the most severe form of the disease. Q188R and K285N mutations are common in Eastern European populations, representing 54–70% of the type 1 galactosemia mutations [8,9,10,11,12].

Other mutations found are S135L, frequent in African-American people, L145P and N314D (the D2 Duarte variant), presented in 5% of the United States population. This N314D mutation is associated with the reduction in enzyme activity, causing a mild form of the disease [8,13,14,15].

In our case, Q188R mutation (c.563A>G [p. Gln188Arg]) was found in a heterozygous state. It is the most frequent mutation in the European population (64%) and can be associated with an important decreased of enzyme activity and a severe phenotype [15]. The second mutation found in our patient (c. 910C>T) is a novel mutation in GALT gene [16].

The clinical manifestations of type 1 galactosemia are secondary to galactose consumption and occurs in the newborn period or in the first few months of life. This is due to the fact that in the first period of life, the only food is milk (breast milk or formula), the main source of galactose. The enzymatic deficiency leads to the accumulation of Gal-1-P, galactose, galactitol or galactonate in hepatocytes, renal tubular cells, neurons, and erythrocytes. At the same time, GALT deficiency inhibits glycogenolysis, causing hypoglycemia and reducing UDP-Galactose, which is the precursor for glycoprotein and glycolipids [2].

The important symptoms are jaundice, hepatosplenomegaly, hepatocellular insufficiency, vomiting, hypoglycemia, hypotonia, seizures, hemolytic anemia, and cataract. A positive urine-reducing substance test, even one not specific to galactosemia, could be another puzzle piece that confirms suspicions of a metabolic disease in infants. The liver is the main affected organ in GALT-deficiency. The early histopathological aspect is represented by hepatocyte fatty degeneration and bile duct proliferation. The prognosis of ALF associated with galactosemia is poor and depends by early diagnosis and stopping galactose intake. In children, the diagnosis of ALF is based on PALF criteria like a syndrome characterized by jaundice, coagulopathy (INR > 1.5) and hepatic encephalopathy in patients with no evidence of prior liver disease. The toxic metabolites also accumulate in the kidneys and central nervous system [17]. If diagnosis is delayed, hepatic injury will progress with possible fatal evolution or fibrotic changes and cirrhosis [2]. The indication for liver transplantation in galactosemia is often difficult, not only because it is a reversible disorder, but also because dysfunction of other organs or systems (MSOF–multisystem organ failure) could be also be associated [18]. Accumulation of galactose and galactitol in lens cells leads to opacity of the lens (“oil droplet” cataract), which progresses to blindness [6]. The incidence of cataracts in patients with galactosemia is 38–75% [2,19]. Also, an excess of galactoso-1-phosphat and other toxic metabolites in different tissues causes Fanconi syndrome, delayed development, speech difficulties, intellectual disability, and ovarian insufficiency. Mental retardation occurs after 6–12 months and is often irreversible [2].

One of the most common causes of early death in children with galactosemia is infection. These patients have an increased risk of sepsis, especially with *Escherichia Coli* [19,20,21]. Other organisms like *Klebsiella*, *Staphylococcus*, *Group B Streptococcus* (GBS), *Streptococcus faecalis* or *Candida* can also be involved. The substrate for the bacterial proliferation is the galactose excess, which inhibits bactericidal activity of polymorphonuclear cells and phagocytosis [21]. The neutrophil function is depressed, favoring bacterial multiplication and severe infections, especially with lactose-fermenting bacteria [22].

*Group B Streptococcus* (GBS or *Streptococcus agalactiae*) is a Gram-positive coccus that frequently colonizes the genital and gastrointestinal tract. The encapsulated coccus can produce toxic polysaccharides, which ensure a high virulence [23]. The GBS infection is rare in healthy children, but is one of the major causes of neonatal sepsis (1–5.4/1000 newborn in USA) [22]. It is associated with invasive infections, especially in immunocompromised hosts. The infection with *Streptococcus agalactiae* in neonate occurs during passage through the vagina, but in some cases, may be vertically, in utero. The newborn is susceptible to infection due to the absence of specific antibodies against GBS [23,24,25]. The manifestations of newborn GBS infection are sepsis (50%), pneumonia (35%), meningitis (15%), skin and soft-tissue infection, osteomyelitis, and septic arthritis [21]. These infections lead to high morbidity and mortality, especially among newborns, premature babies, and children with comorbidities [22].

Galactosemia type II (also called galactokinase deficiency) and type III (galactose epimerase deficiency) have different patterns of signs and symptoms. Galactosemia type II (GALK deficiency) causes fewer medical problems than the classic type. Affected infants develop cataract or pseudotumor cerebri later. Galactosemia type II is secondary to galactokinase deficiency, the enzyme involved in the transformation of galactose into galactose 1 phosphate. The galactose excess is metabolized by an alternative method, resulting in galactonic acid and galactitol, which is responsible for cataract. The signs and symptoms of GALE deficiency (galactosemia type III) vary from mild to severe and can include cataract, delayed growth or development, intellectual disability, liver disease, and kidney problems [23].

## 4. Conclusions

We presented a rare association between two extremely severe diseases in neonates: *Group B Streptococcus* (GBS) meningitis/sepsis and acute liver failure (ALF), both requiring prompt diagnosis and specific treatment, in a patient with late diagnosed galactosemia, as consequences of this metabolic injury. Genetic tests revealed a compound heterozygous status with Q188R and a novel mutation in the GALT gene. If a galactose-free diet is initiated during the first days of life, the prognosis is significantly improved and the evolution to acute liver failure, sepsis or the other complications can be prevented. In developed countries, the diagnosis of galactosemia is made early, by extensive newborn screening programs. Unfortunately, in Romania, the neonatal screening program does not include galactosemia, but clinical recognition and rapid urine/blood samples collection (before supportive therapy), followed by an appropriate test and a lactose-free diet prevents severe consequences.
